# *trans*-Diaqua­bis[5-carb­oxy-2-(3-pyridyl)-1*H*-imidazole-4-carboxyl­ato-κ^2^ *N* ^3^,*O* ^4^]manganese(II)

**DOI:** 10.1107/S1600536808029073

**Published:** 2008-09-20

**Authors:** Li-Zhuang Chen

**Affiliations:** a Ordered Matter Science Research Center, College of Chemistry and Chemical Engineering, Southeast University, Nanjing 210096, People’s Republic of China

## Abstract

In the title compound, [Mn(C_10_H_6_N_3_O_4_)_2_(H_2_O)_2_], synthesized by hydro­thermal reaction, the Mn^II^ ion lies on an inversion centre and displays a distorted octa­hedral coordination geometry defined by the two imidazole N atoms and two carboxylate O atoms of the two *trans*-standing chelate ligands, and two O atoms of the water mol­ecules. A two-dimensional supra­molecular architecture is formed *via* N—H⋯O, O—H⋯N and O—H⋯O hydrogen-bonding inter­actions.

## Related literature

For the chemistry of imidazoles, see: Xiao *et al.* (2004 ▶); Zhang *et al.* (2004 ▶); Lu *et al.* (2006 ▶).
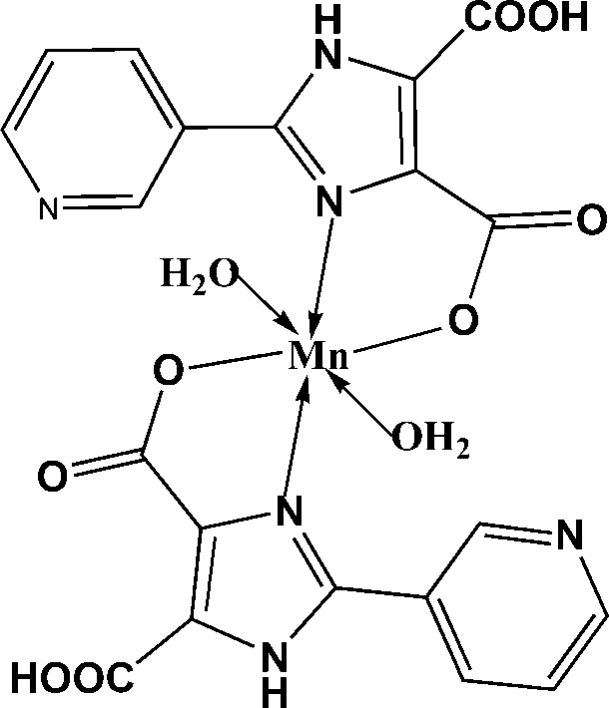


## Experimental

### 

#### Crystal data

[Mn(C_10_H_6_N_3_O_4_)_2_(H_2_O)_2_]
*M* *_r_* = 555.33
Triclinic, 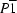

*a* = 6.9574 (7) Å
*b* = 8.5636 (7) Å
*c* = 9.4409 (16) Å
α = 81.90 (3)°
β = 83.42 (4)°
γ = 72.10 (2)°
*V* = 528.41 (11) Å^3^
*Z* = 1
Mo *K*α radiation
μ = 0.70 mm^−1^
*T* = 298 (2) K
0.25 × 0.20 × 0.20 mm

#### Data collection

Rigaku Mercury2 diffractometer
Absorption correction: multi-scan (*CrystalClear*; Rigaku, 2005 ▶) *T*_min_ = 0.845, *T*_max_ = 0.869
5416 measured reflections
2378 independent reflections
1871 reflections with *I* > 2σ(*I*)
*R*_int_ = 0.029

#### Refinement

*R*[*F* ^2^ > 2σ(*F* ^2^)] = 0.047
*wR*(*F* ^2^) = 0.165
*S* = 1.11
2378 reflections
175 parameters
2 restraints
H atoms treated by a mixture of independent and constrained refinement
Δρ_max_ = 0.54 e Å^−3^
Δρ_min_ = −0.44 e Å^−3^

### 

Data collection: *CrystalClear* (Rigaku, 2005 ▶); cell refinement: *CrystalClear*; data reduction: *CrystalClear*; program(s) used to solve structure: *SHELXS97* (Sheldrick, 2008 ▶); program(s) used to refine structure: *SHELXL97* (Sheldrick, 2008 ▶); molecular graphics: *SHELXTL* (Sheldrick, 2008 ▶); software used to prepare material for publication: *SHELXTL*.

## Supplementary Material

Crystal structure: contains datablocks I, global. DOI: 10.1107/S1600536808029073/bx2177sup1.cif

Structure factors: contains datablocks I. DOI: 10.1107/S1600536808029073/bx2177Isup2.hkl

Additional supplementary materials:  crystallographic information; 3D view; checkCIF report

## Figures and Tables

**Table 1:** Hydrogen-bond geometry (Å, °)

*D*—H⋯*A*	*D*—H	H⋯*A*	*D*⋯*A*	*D*—H⋯*A*
N2—H2*A*⋯O4^i^	0.86	2.00	2.840 (3)	164
O5—H5⋯N3^ii^	0.82	1.97	2.779 (3)	171
O5—H5*B*⋯O3^iii^	0.839 (17)	2.074 (18)	2.908 (3)	173 (3)
O3—H3⋯O2	0.82	1.69	2.456 (3)	155

Symmetry codes: (i) 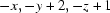; (ii) 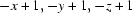; (iii) 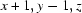.

**Fig. 1.**
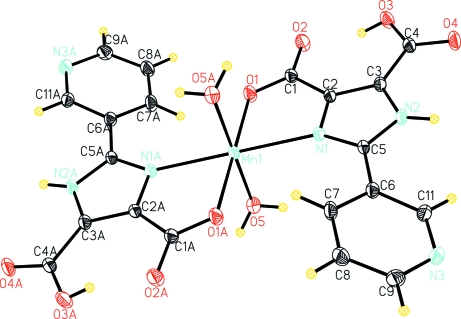
A view of the title compound with the atomic numbering scheme. Displacement ellipsoids were drawn at the 30% probability level and H atoms are shown as small spheres of arbitrary radii. Unlabelled atoms are related to labelled atoms by the symmetry code ( -x+1, -y+1, -z).

**Fig. 2.**
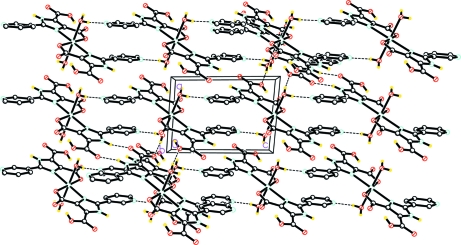
The crystal packing of the title compound viewed along the b axis and all hydrogen atoms not involved in hydrogen bonding (dashed lines) were omitted for clarity.

## supplementary crystallographic information

### Comment

N-Heterocyclic carboxylic acids, such as imidazole-4,5-dicarboxylic acid, are recognized as efficient N,*O*-donors, exhibiting diverse modes of coordination (Zhang *et al.*, 2004; Xiao *et al.*, 2004; Lu *et al.*, 2006). In this work, we have chosen 2-Pyridin-3-yl-1*H*-imidazole-4,5-dicarboxylic acid as the building block to obtain the title compound, and we present its crystal structure here. Mn^II^ ion lies on an inversion centre and displaying distorted octahedral coordination geometry defined by the two imidazole N atoms, two O toms of the carboxylate groups and two O atoms of the water molecules. The pyridine ring and imidazole rings are twisted from each other by a dihedral angle of 20.78 (2)° (Fig. 1). The crystal structure is stabilized by intermolecular O—H···N, O—H···O and N—H···O, hydrogen bonds. A two-dimensional supramolecular architecture is formed *via* hydrogen-bond interactions (Table 1 and Fig. 2).

### Experimental

A mixture of 2-Pyridin-3-yl-1*H*-imidazole-4,5-dicarboxylic acid (0.1 mmol, 23 mg) and MnCl_2_ (20 mg, 0.1 mmol) and water (1 ml) sealed in a glass tube was maintained at 100°C for 3 d then cooled to room temperature to obtain suitable single crystals for X-ray analysis.

### Refinement

All H atoms attached to C atoms, O atoms and N atoms except H5B were fixed geometrically and treated as riding with C—H = 0.93 Å (aromatic), O—H = 0.82 Å and N—H = 0.86 Å with *U*_iso_(H) = 1.2*U*_eq_(C and N) or *U*_iso_(H) = 1.5*U*_eq_(O). H5B atom of H_2_O were located in difference Fourier maps.

### Crystal data

**Table:**

[Mn(C_10_H_6_N_3_O_4_)_2_(H_2_O)_2_]	*Z* = 1
*M**_r_* = 555.33	*F*(000) = 283
Triclinic, *P*1	*D*_x_ = 1.745 Mg m^−^^3^
Hall symbol: -P 1	Mo *K*α radiation, λ = 0.71073 Å
*a* = 6.9574 (7) Å	Cell parameters from 1463 reflections
*b* = 8.5636 (7) Å	θ = 3.1–27.5°
*c* = 9.4409 (16) Å	µ = 0.70 mm^−^^1^
α = 81.90 (3)°	*T* = 298 K
β = 83.42 (4)°	Block, colourless
γ = 72.10 (2)°	0.25 × 0.20 × 0.20 mm
*V* = 528.41 (11) Å^3^	

### Data collection

**Table:**

Rigaku Mercury2 diffractometer	2378 independent reflections
Radiation source: fine-focus sealed tube	1871 reflections with *I* > 2σ(*I*)
graphite	*R*_int_ = 0.029
Detector resolution: 13.6612 pixels mm^-1^	θ_max_ = 27.5°, θ_min_ = 2.5°
ω scans	*h* = −8→8
Absorption correction: multi-scan (CrystalClear; Rigaku, 2005)	*k* = −11→11
*T*_min_ = 0.845, *T*_max_ = 0.869	*l* = −12→12
5416 measured reflections	

### Refinement

**Table:**

Refinement on *F*^2^	Primary atom site location: structure-invariant direct methods
Least-squares matrix: full	Secondary atom site location: difference Fourier map
*R*[*F*^2^ > 2σ(*F*^2^)] = 0.047	Hydrogen site location: inferred from neighbouring sites
*wR*(*F*^2^) = 0.165	H atoms treated by a mixture of independent and constrained refinement
*S* = 1.11	*w* = 1/[σ^2^(*F*_o_^2^) + (0.1003*P*)^2^ + 0.0122*P*] where *P* = (*F*_o_^2^ + 2*F*_c_^2^)/3
2378 reflections	(Δ/σ)_max_ < 0.001
175 parameters	Δρ_max_ = 0.54 e Å^−^^3^
2 restraints	Δρ_min_ = −0.44 e Å^−^^3^

### Special details

**Table:**

Geometry. All e.s.d.'s (except the e.s.d. in the dihedral angle between two l.s. planes) are estimated using the full covariance matrix. The cell e.s.d.'s are taken into account individually in the estimation of e.s.d.'s in distances, angles and torsion angles; correlations between e.s.d.'s in cell parameters are only used when they are defined by crystal symmetry. An approximate (isotropic) treatment of cell e.s.d.'s is used for estimating e.s.d.'s involving l.s. planes.
Refinement. Refinement of *F*^2^ against ALL reflections. The weighted *R*-factor *wR* and goodness of fit *S* are based on *F*^2^, conventional *R*-factors *R* are based on *F*, with *F* set to zero for negative *F*^2^. The threshold expression of *F*^2^ > σ(*F*^2^) is used only for calculating *R*-factors(gt) *etc*. and is not relevant to the choice of reflections for refinement. *R*-factors based on *F*^2^ are statistically about twice as large as those based on *F*, and *R*- factors based on ALL data will be even larger.

### Fractional atomic coordinates and isotropic or equivalent isotropic displacement parameters (Å^2^)

**Table:**

	*x*	*y*	*z*	*U*_iso_*/*U*_eq_	
Mn1	0.5000	0.5000	0.0000	0.0299 (2)	
C1	0.3266 (4)	0.8677 (4)	−0.0160 (3)	0.0290 (6)	
C2	0.2634 (4)	0.8148 (3)	0.1332 (3)	0.0253 (6)	
C3	0.1577 (4)	0.9082 (3)	0.2405 (3)	0.0260 (6)	
C4	0.0487 (5)	1.0861 (4)	0.2425 (3)	0.0308 (6)	
C5	0.2472 (4)	0.6428 (3)	0.3206 (3)	0.0229 (5)	
C6	0.2698 (4)	0.4926 (3)	0.4203 (3)	0.0253 (6)	
C7	0.3049 (5)	0.3401 (4)	0.3713 (3)	0.0336 (7)	
H7A	0.3120	0.3314	0.2737	0.040*	
C8	0.3291 (5)	0.2013 (4)	0.4703 (3)	0.0369 (7)	
H8A	0.3592	0.0972	0.4399	0.044*	
C9	0.3080 (5)	0.2195 (4)	0.6156 (3)	0.0345 (7)	
H9A	0.3215	0.1261	0.6817	0.041*	
N3	0.2692 (4)	0.3661 (3)	0.6640 (3)	0.0332 (6)	
C11	0.2491 (5)	0.4986 (4)	0.5688 (3)	0.0300 (6)	
H11A	0.2196	0.6010	0.6025	0.036*	
N1	0.3172 (3)	0.6504 (3)	0.1834 (2)	0.0255 (5)	
N2	0.1531 (4)	0.7971 (3)	0.3573 (2)	0.0269 (5)	
H2A	0.0992	0.8207	0.4412	0.032*	
O1	0.4330 (4)	0.7595 (3)	−0.0912 (2)	0.0380 (5)	
O2	0.2722 (4)	1.0220 (3)	−0.0599 (2)	0.0408 (6)	
O3	0.0641 (4)	1.1816 (3)	0.1292 (2)	0.0397 (6)	
H3	0.1602	1.1348	0.0764	0.060*	
O4	−0.0526 (4)	1.1310 (3)	0.3540 (2)	0.0457 (6)	
O5	0.7793 (4)	0.5087 (3)	0.0738 (2)	0.0414 (6)	
H5	0.7544	0.5539	0.1476	0.062*	
H5B	0.867 (5)	0.416 (3)	0.083 (3)	0.042 (10)*	

### Atomic displacement parameters (Å^2^)

**Table:**

	*U*^11^	*U*^22^	*U*^33^	*U*^12^	*U*^13^	*U*^23^
Mn1	0.0369 (4)	0.0247 (4)	0.0204 (3)	0.0026 (3)	0.0007 (3)	−0.0056 (2)
C1	0.0337 (15)	0.0254 (14)	0.0204 (13)	0.0004 (11)	0.0015 (11)	−0.0017 (10)
C2	0.0307 (14)	0.0207 (13)	0.0197 (13)	−0.0006 (10)	−0.0005 (11)	−0.0035 (10)
C3	0.0315 (14)	0.0231 (14)	0.0205 (12)	−0.0040 (11)	0.0015 (11)	−0.0049 (10)
C4	0.0366 (16)	0.0242 (14)	0.0257 (14)	0.0005 (12)	−0.0005 (12)	−0.0061 (11)
C5	0.0233 (13)	0.0235 (13)	0.0184 (12)	−0.0021 (10)	0.0020 (10)	−0.0047 (9)
C6	0.0270 (13)	0.0265 (14)	0.0208 (13)	−0.0063 (11)	0.0028 (11)	−0.0041 (10)
C7	0.0455 (17)	0.0288 (15)	0.0212 (14)	−0.0052 (13)	0.0077 (13)	−0.0064 (11)
C8	0.0493 (19)	0.0225 (14)	0.0336 (16)	−0.0033 (12)	−0.0003 (14)	−0.0048 (11)
C9	0.0404 (17)	0.0279 (15)	0.0303 (16)	−0.0074 (13)	0.0003 (13)	0.0039 (12)
N3	0.0419 (15)	0.0327 (13)	0.0209 (12)	−0.0075 (11)	0.0023 (10)	−0.0012 (9)
C11	0.0388 (16)	0.0271 (14)	0.0226 (14)	−0.0065 (12)	−0.0030 (12)	−0.0041 (11)
N1	0.0310 (12)	0.0214 (11)	0.0200 (11)	−0.0021 (9)	−0.0007 (10)	−0.0028 (9)
N2	0.0334 (12)	0.0246 (12)	0.0185 (11)	−0.0032 (10)	0.0038 (9)	−0.0058 (8)
O1	0.0503 (14)	0.0304 (11)	0.0210 (10)	0.0023 (10)	0.0077 (9)	−0.0028 (8)
O2	0.0578 (14)	0.0251 (11)	0.0257 (11)	0.0011 (10)	0.0082 (10)	0.0035 (8)
O3	0.0508 (14)	0.0229 (11)	0.0330 (12)	0.0038 (9)	0.0059 (10)	−0.0031 (8)
O4	0.0650 (16)	0.0303 (12)	0.0291 (12)	0.0059 (11)	0.0040 (11)	−0.0123 (9)
O5	0.0423 (13)	0.0434 (14)	0.0309 (12)	0.0012 (11)	−0.0015 (10)	−0.0119 (10)

### Geometric parameters (Å, °)

**Table:**

Mn1—O5^i^	2.163 (2)	C5—N2	1.356 (3)
Mn1—O5	2.163 (2)	C5—C6	1.460 (4)
Mn1—O1^i^	2.194 (2)	C6—C7	1.389 (4)
Mn1—O1	2.194 (2)	C6—C11	1.399 (4)
Mn1—N1^i^	2.322 (2)	C7—C8	1.383 (4)
Mn1—N1	2.322 (2)	C7—H7A	0.9300
C1—O1	1.246 (3)	C8—C9	1.388 (4)
C1—O2	1.279 (3)	C8—H8A	0.9300
C1—C2	1.477 (4)	C9—N3	1.335 (4)
C2—N1	1.370 (3)	C9—H9A	0.9300
C2—C3	1.380 (4)	N3—C11	1.326 (4)
C3—N2	1.355 (3)	C11—H11A	0.9300
C3—C4	1.480 (4)	N2—H2A	0.8600
C4—O4	1.238 (3)	O3—H3	0.8200
C4—O3	1.267 (4)	O5—H5	0.8200
C5—N1	1.331 (3)	O5—H5B	0.839 (17)
			
O5^i^—Mn1—O5	180.00 (11)	N2—C5—C6	123.9 (2)
O5^i^—Mn1—O1^i^	90.51 (10)	C7—C6—C11	117.7 (3)
O5—Mn1—O1^i^	89.49 (10)	C7—C6—C5	121.3 (2)
O5^i^—Mn1—O1	89.49 (10)	C11—C6—C5	121.0 (2)
O5—Mn1—O1	90.51 (10)	C8—C7—C6	118.9 (3)
O1^i^—Mn1—O1	180.0	C8—C7—H7A	120.6
O5^i^—Mn1—N1^i^	90.00 (8)	C6—C7—H7A	120.6
O5—Mn1—N1^i^	90.00 (8)	C7—C8—C9	119.2 (3)
O1^i^—Mn1—N1^i^	74.86 (8)	C7—C8—H8A	120.4
O1—Mn1—N1^i^	105.14 (8)	C9—C8—H8A	120.4
O5^i^—Mn1—N1	90.00 (8)	N3—C9—C8	122.5 (3)
O5—Mn1—N1	90.00 (8)	N3—C9—H9A	118.8
O1^i^—Mn1—N1	105.14 (8)	C8—C9—H9A	118.8
O1—Mn1—N1	74.86 (8)	C11—N3—C9	118.2 (3)
N1^i^—Mn1—N1	180.0	N3—C11—C6	123.5 (3)
O1—C1—O2	123.7 (3)	N3—C11—H11A	118.2
O1—C1—C2	118.1 (3)	C6—C11—H11A	118.2
O2—C1—C2	118.2 (3)	C5—N1—C2	105.7 (2)
N1—C2—C3	110.3 (2)	C5—N1—Mn1	145.47 (18)
N1—C2—C1	119.8 (2)	C2—N1—Mn1	108.75 (16)
C3—C2—C1	129.9 (3)	C3—N2—C5	109.1 (2)
N2—C3—C2	104.8 (2)	C3—N2—H2A	125.5
N2—C3—C4	121.9 (2)	C5—N2—H2A	125.5
C2—C3—C4	133.1 (3)	C1—O1—Mn1	118.36 (18)
O4—C4—O3	124.5 (3)	C4—O3—H3	109.5
O4—C4—C3	117.8 (3)	Mn1—O5—H5	109.5
O3—C4—C3	117.7 (2)	Mn1—O5—H5B	113 (2)
N1—C5—N2	110.0 (2)	H5—O5—H5B	112.4
N1—C5—C6	126.0 (2)		
			
O1—C1—C2—N1	−1.9 (4)	C6—C5—N1—C2	−179.2 (3)
O2—C1—C2—N1	179.4 (3)	N2—C5—N1—Mn1	176.6 (2)
O1—C1—C2—C3	175.6 (3)	C6—C5—N1—Mn1	−2.2 (5)
O2—C1—C2—C3	−3.1 (5)	C3—C2—N1—C5	−0.6 (3)
N1—C2—C3—N2	1.4 (3)	C1—C2—N1—C5	177.3 (3)
C1—C2—C3—N2	−176.2 (3)	C3—C2—N1—Mn1	−178.84 (19)
N1—C2—C3—C4	−173.1 (3)	C1—C2—N1—Mn1	−0.9 (3)
C1—C2—C3—C4	9.3 (6)	O5^i^—Mn1—N1—C5	95.6 (3)
N2—C3—C4—O4	−1.9 (5)	O5—Mn1—N1—C5	−84.4 (3)
C2—C3—C4—O4	171.9 (3)	O1^i^—Mn1—N1—C5	5.0 (4)
N2—C3—C4—O3	178.8 (3)	O1—Mn1—N1—C5	−175.0 (4)
C2—C3—C4—O3	−7.4 (5)	O5^i^—Mn1—N1—C2	−87.49 (19)
N1—C5—C6—C7	−22.7 (4)	O5—Mn1—N1—C2	92.51 (19)
N2—C5—C6—C7	158.6 (3)	O1^i^—Mn1—N1—C2	−178.01 (18)
N1—C5—C6—C11	159.7 (3)	O1—Mn1—N1—C2	1.99 (18)
N2—C5—C6—C11	−18.9 (4)	C2—C3—N2—C5	−1.7 (3)
C11—C6—C7—C8	−3.7 (4)	C4—C3—N2—C5	173.6 (3)
C5—C6—C7—C8	178.7 (3)	N1—C5—N2—C3	1.4 (3)
C6—C7—C8—C9	3.1 (5)	C6—C5—N2—C3	−179.8 (2)
C7—C8—C9—N3	−1.4 (5)	O2—C1—O1—Mn1	−177.4 (2)
C8—C9—N3—C11	0.4 (5)	C2—C1—O1—Mn1	3.9 (4)
C9—N3—C11—C6	−1.1 (5)	O5^i^—Mn1—O1—C1	86.8 (2)
C7—C6—C11—N3	2.8 (4)	O5—Mn1—O1—C1	−93.2 (2)
C5—C6—C11—N3	−179.6 (3)	N1^i^—Mn1—O1—C1	176.7 (2)
N2—C5—N1—C2	−0.4 (3)	N1—Mn1—O1—C1	−3.3 (2)

Symmetry codes: (i) −*x*+1, −*y*+1, −*z*.

### Hydrogen-bond geometry (Å, °)

**Table:**

*D*—H···*A*	*D*—H	H···*A*	*D*···*A*	*D*—H···*A*
N2—H2A···O4^ii^	0.86	2.00	2.840 (3)	164.
O5—H5···N3^iii^	0.82	1.97	2.779 (3)	171.
O5—H5B···O3^iv^	0.84 (2)	2.07 (2)	2.908 (3)	173 (3)
O3—H3···O2	0.82	1.69	2.456 (3)	155.

Symmetry codes: (ii) −*x*, −*y*+2, −*z*+1; (iii) −*x*+1, −*y*+1, −*z*+1; (iv) *x*+1, *y*−1, *z*.
